# Supplementary material for: Iron commensalism of mesenchymal glioblastoma promotes ferroptosis susceptibility upon dopamine treatment
Source: Commun Biol. 2022 Jun 16;5:593. doi: 10.1038/s42003-022-03538-y (PMC9203457; doi:10.1038/s42003-022-03538-y)
Supplement: Supplementary file 2 — Supplementary Information [file 42003_2022_3538_MOESM2_ESM.pdf]

Supplementary Information

Iron commensalism of mesenchymal glioblastoma promotes ferroptosis susceptibility upon dopamine treatment.

Vu T. A. Vo[1,2,3], Sohyun Kim[4], Tuyen N. M. Hua[1,3], Jiwoong Oh[5], Yangsik Jeong[1,2,3,6,7,*]

Departments of [1]Biochemistry, [2]Global Medical Science, and [3]Mitohormesis Research Center, Wonju College of Medicine, Yonsei University, Wonju, Gangwon-do, Republic of Korea

[4]Department of Physiology, Yonsei University College of Medicine, and [5]Department of Neurosurgery, Severance Hospital, Yonsei University, Seoul, Republic of Korea

Institutes of [6]Lifestyle Medicine and [7]Mitochondrial Medicine, Wonju College of Medicine, Yonsei University, Wonju, Gangwon-do, Republic of Korea

[*]Corresponding author.

Yangsik Jeong

Department of Biochemistry, Wonju College of Medicine, Yonsei University, 20 Ilsan, Wonju, Gangwon-do, 26426, Republic of Korea

Tel: +82-33-741-0284

Fax: +82-33-743-0411

Email: yjeong@yonsei.ac.kr

Supplementary information contains 9 figures and 1 table.

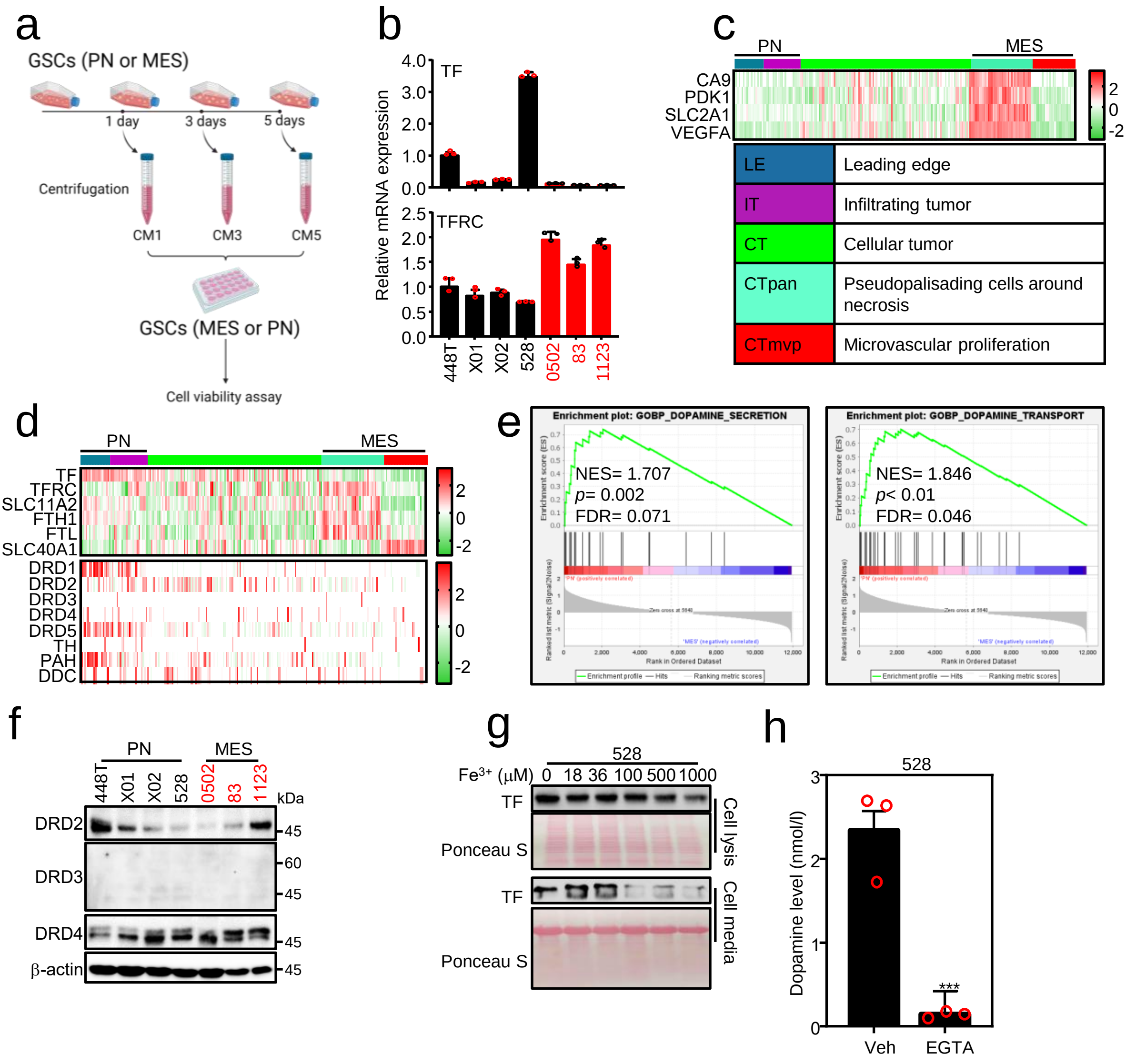

Supplementary Figure 1. PN GSCs secrete DA and TF.

(a) Scheme of the experimental plan, figure created with BioRender.com. (b) mRNA expression of *TF* and *TFRC* in the GSC panel determined via quantitative real-time PCR (qPCR). (c, d) Expression profile for genes of interest from the Ivy GAP database. Expression pattern for hypoxia-associated genes (c) and genes (d) involved in the metabolism of iron or related to DA in anatomically microdissected regions of GBM tissues. The microdissected regions are annotated in (c). (e) GSEA for enriched pathway in PN and MES regions of Ivy GAP database. (f) Protein expression of DRD2, 3, and 4 in the GSC panel. (g) Expression of TF in cell lysis and cell media of PN 528 upon iron treatment. (h) DA level in cell media of PN 528 upon EGTA treatment.

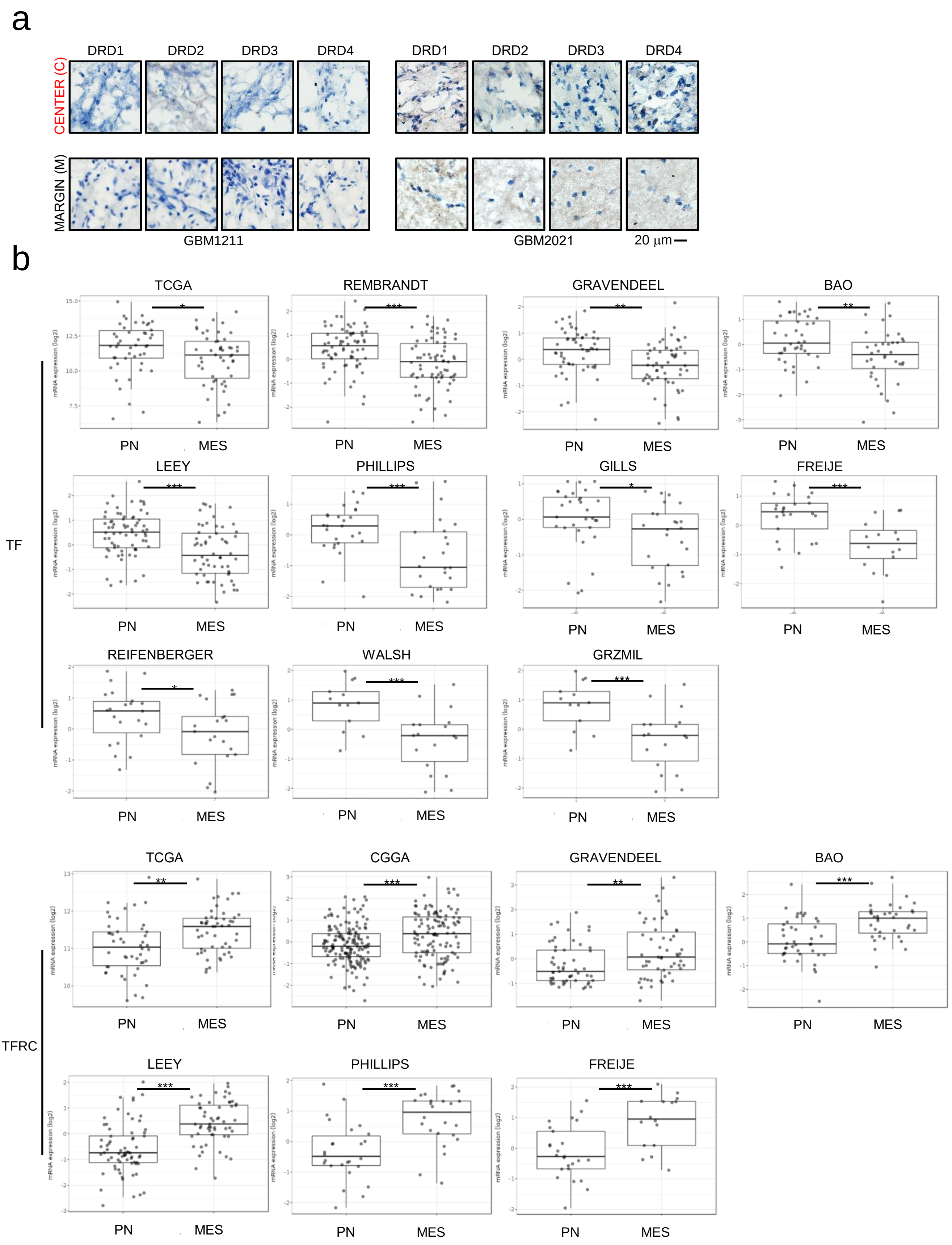

Legend was shown next page

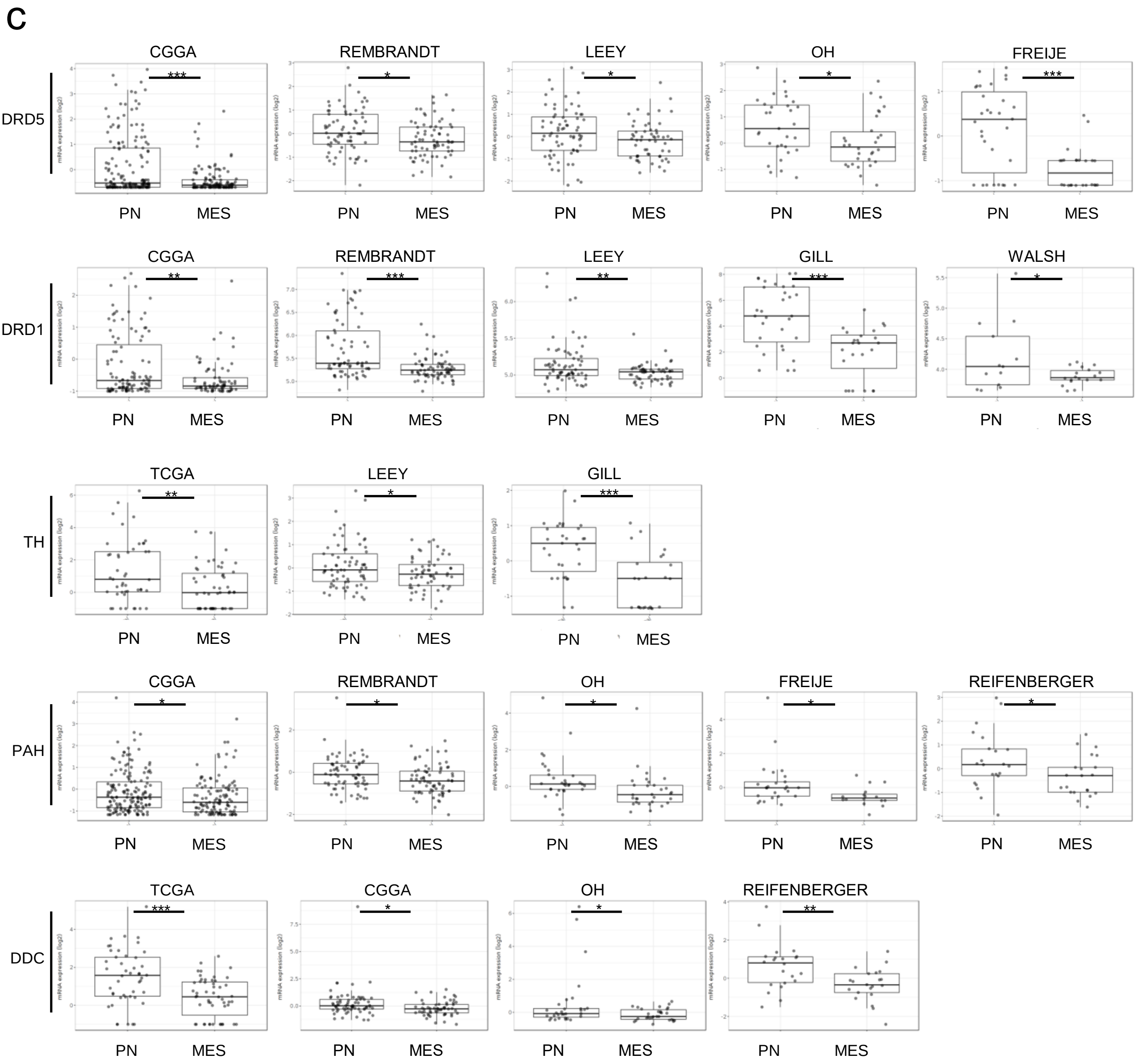

Supplementary Figure 2. (a) Expression of DRD1, 2, 3, and 4 in the GBM tissue samples presented in Fig. 2. (b,c) Expression of (b) iron and (c) DA-related genes in multiple datasets. Data for each gene was analyzed by Gliovis project (http://gliovis.bioinfo.cnio.es/)

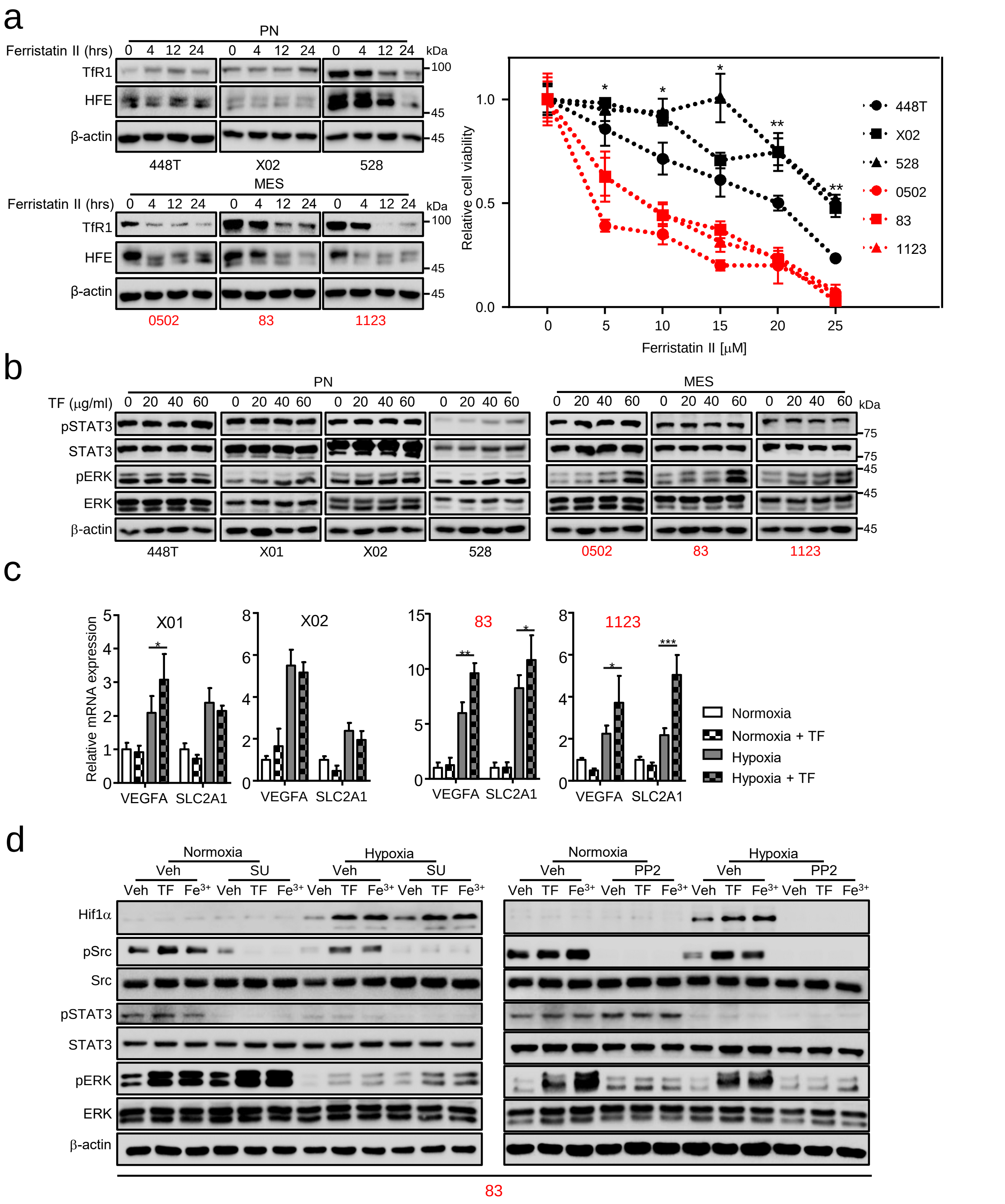

Legend was shown next page

Supplementary Figure 3. TF induces MES GSC proliferation via preferential iron uptake.

(a) Growth response of the GSC panel upon ferristatin II treatment. (left) Time-dependent expression of TfR1 and HFE in the GSC panel following treatment with 25 μM of ferristatin II. (right) Both PN and MES GSCs were assayed for growth response using MTS assays following treatment with different doses of ferristatin II. (b) Protein expression of pSTAT3, STAT3, pERK, and ERK in the GSC panel following treatment with different concentrations of TF. (c) mRNA expression of VEGFA and SLC2A1 in the GSC panel under TF treatment under normoxia or hypoxia. GSCs were treated with 20 μg/ml TF under normoxic or hypoxic conditions for 24 hours and followed by qPCR to determine gene expression. (d) Expression of Hif1α and Src downstreams under normoxic and hypoxic condition with or without treatment of 20 μg/ml TF, 18 μM of Fe^{3+}, 5 μM of SU6656, or 20 μM of PP2.

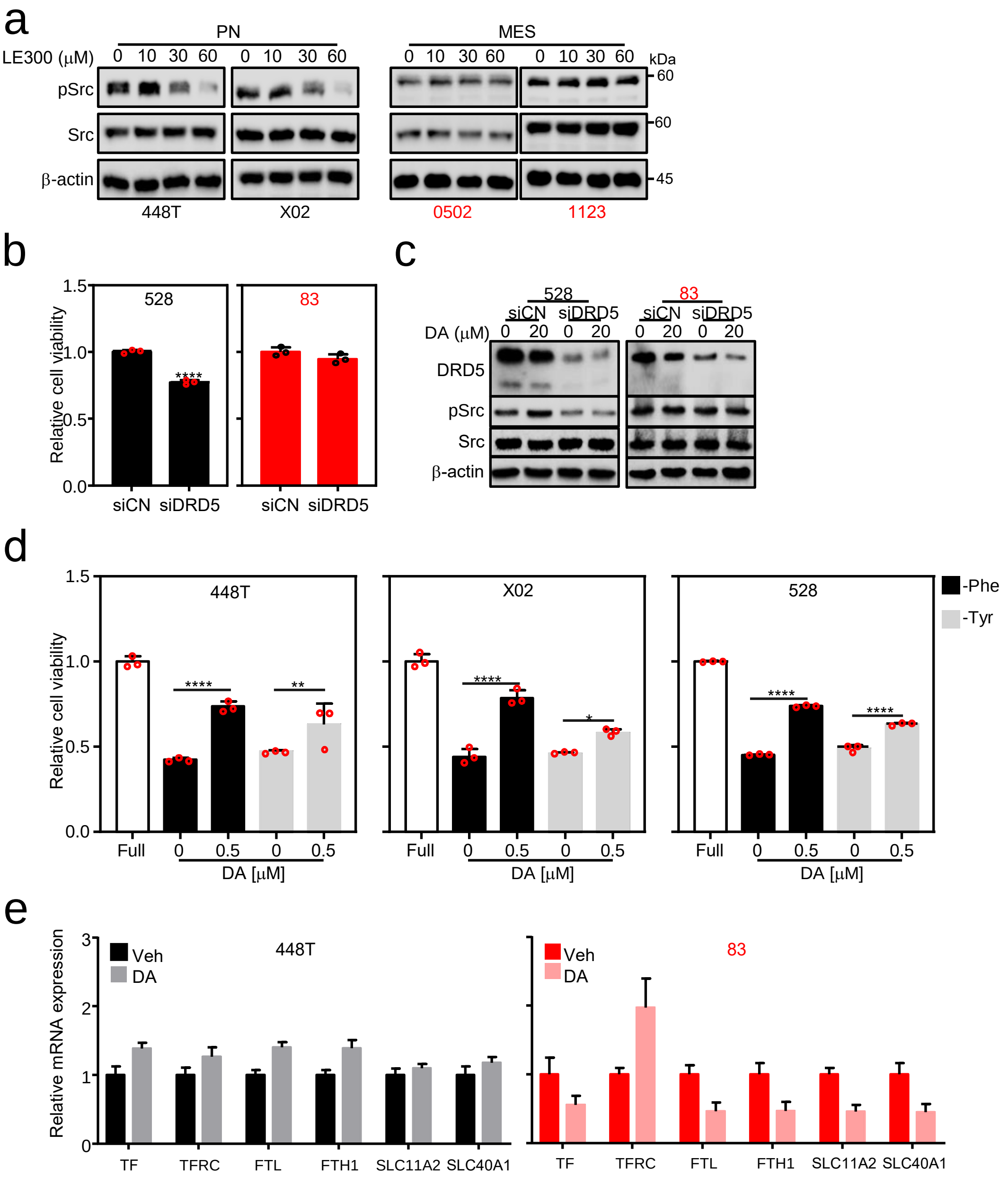

Supplementary Figure 4. DA functions in an autocrine and paracrine manner in GSCs.

(a) Immunoblot of pSrc and Src in the GSC panel. The GSC panel was treated with LE300 at different doses, followed by western blotting for the detection of protein levels. (b) Cell viability of PN 528 and MES 83 upon DRD5 knockdown for 3 days. Cell viability was assayed by MTS. (c) Expression of pSrc upon DRD5 knockdown for 3 days. (d) Addition of DA rescued the reduced PN GSC growth under phenylalanine or tyrosine deprivation condition. The viability of PN GSCs was assessed via MTS assays at 3 days after treatment of DA in phenylalanine or tyrosine deficient media for 3 days. (e) mRNA expression of genes of interest associated with iron metabolism in PN X02 and MES 83 GSCs.

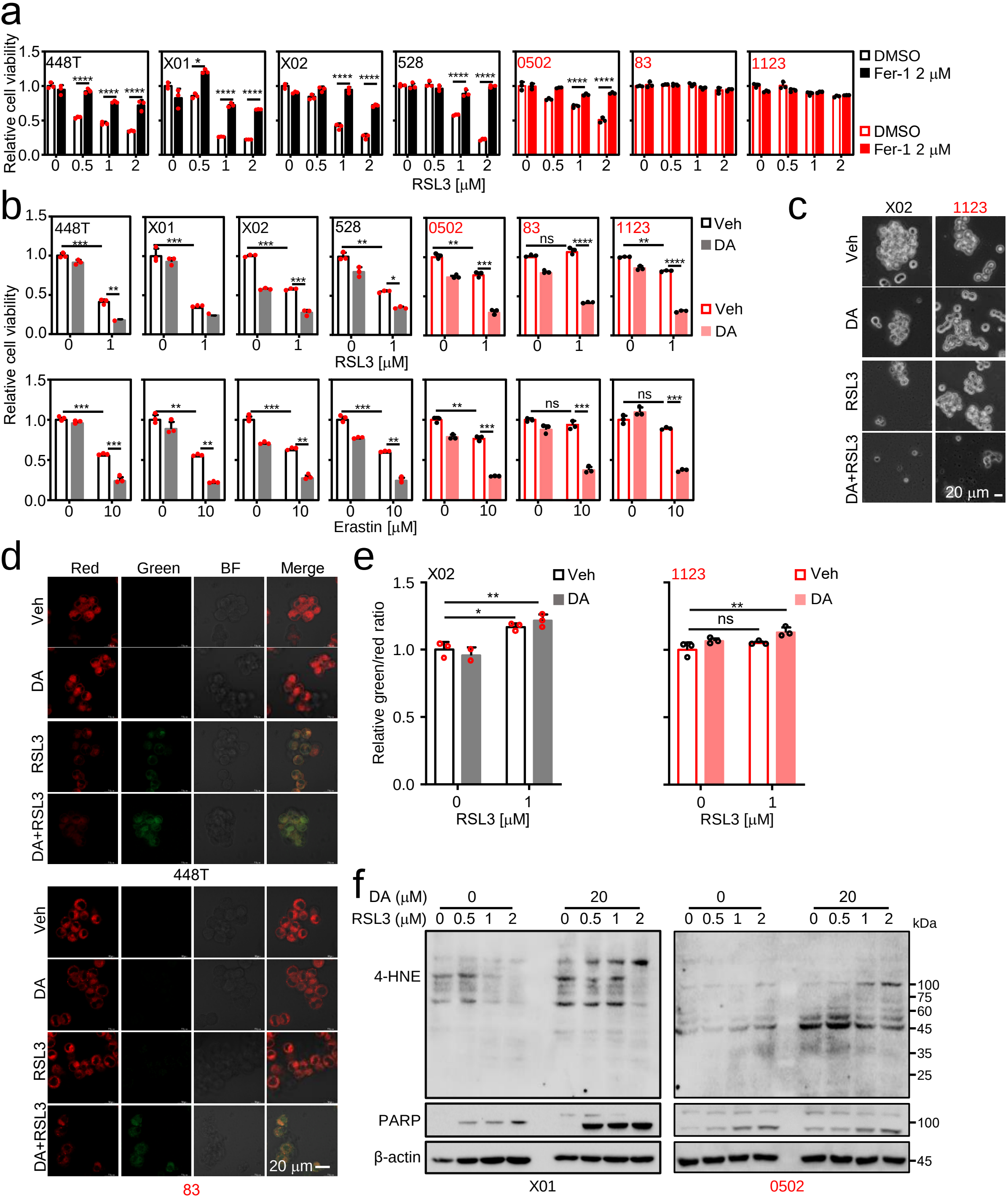

Legend was shown next page

Vo et al.

Supplementary Figure 5. DA enhances ferroptosis in GSCs.

(a, b) Ferroptosis susceptibility of GSCs under various treatment conditions. The viability of GSCs was assessed via MTS assay at 1 day after treatment with the indicated drug combination. Drugs included RSL3 as a GPX4 inhibitor, erastin for inhibiting system Xc, and ferrostatin-1 as a ferroptosis blocker. Cells were treated with 20 μM DA in combination of RSL3 or erastin in (b). (c) Images of PN X02 and MES 1123 GSCs following drug treatment for 1 day. (d-f) DA treatment increased lipid peroxidation. (d) PN 448T and MES 83 GSCs were treated with 1 μM of RSL3 in the presence or absence of 20 μM DA and visualized with BODIPY™ 581/591 C11 incubation. (e) Lipid peroxidation quantification in PN X02 and MES 1123. (f) Immunoblotting for 4-HNE in PN X01 and MES 0502.

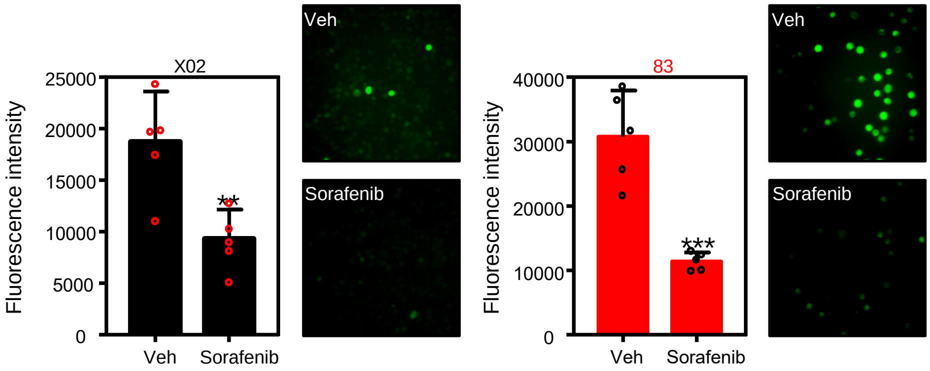

Supplementary Figure 6. Sorafenib reduced cystine uptake in GSCs. GSCs were treated with sorafenib before measuring for cystine uptake representing by fluorescence signal.

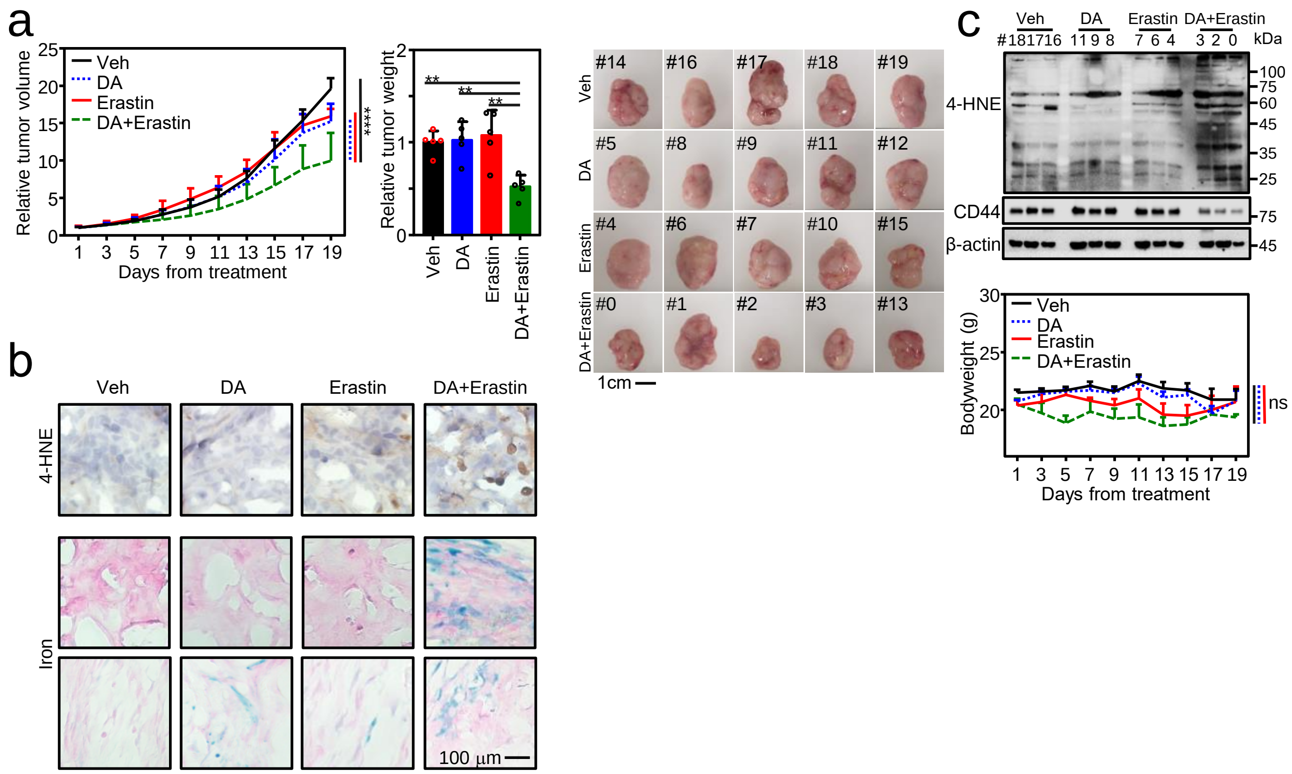

Supplementary Figure 7. DA enhances ferroptosis in GSCs in heterotopic xenograft model.

Tumor regression in the heterotopic xenograft model. (a) Xenografted tumors were monitored by measuring volume (left) during the experiment, and weight (middle, right) at the end of the experiment. (b) Representative tumor tissues from each group were stained for 4-HNE (upper) or intratumoral iron accumulation (lower). (c) Immunoblots of 4-HNE and CD44 in tumor tissues (upper). Mean body weight of each group during treatment (lower).

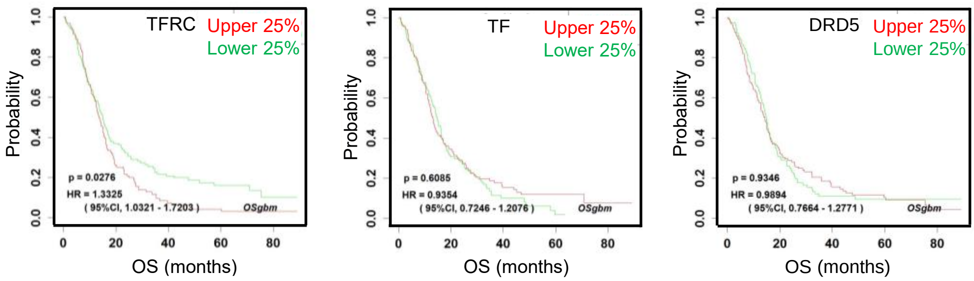

Supplementary Figure 8. Kaplan-Meier's plot of GBM patients based on single gene expression. Prognosis of GBM patients based on *TFRC, TF, DRD5* expression from datasets (GSE30472, GSE42669, GSE4412, GSE7696, Chinese Glioma Genome Atlas (CCGA), and TCGA (Nature 2008)).

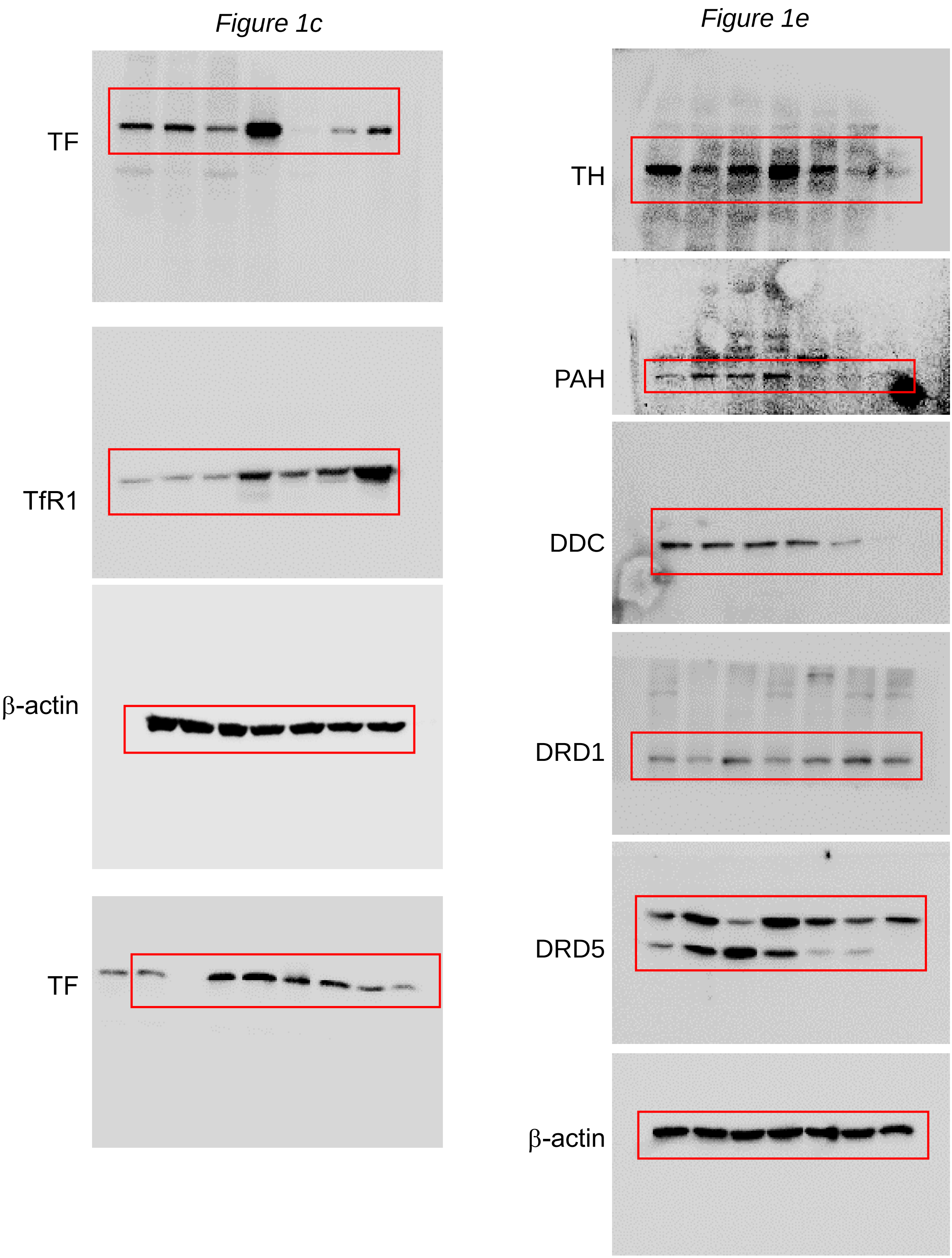

Supplementary Figure 9. Uncropped blots for immunoblot assay

Figure 3c

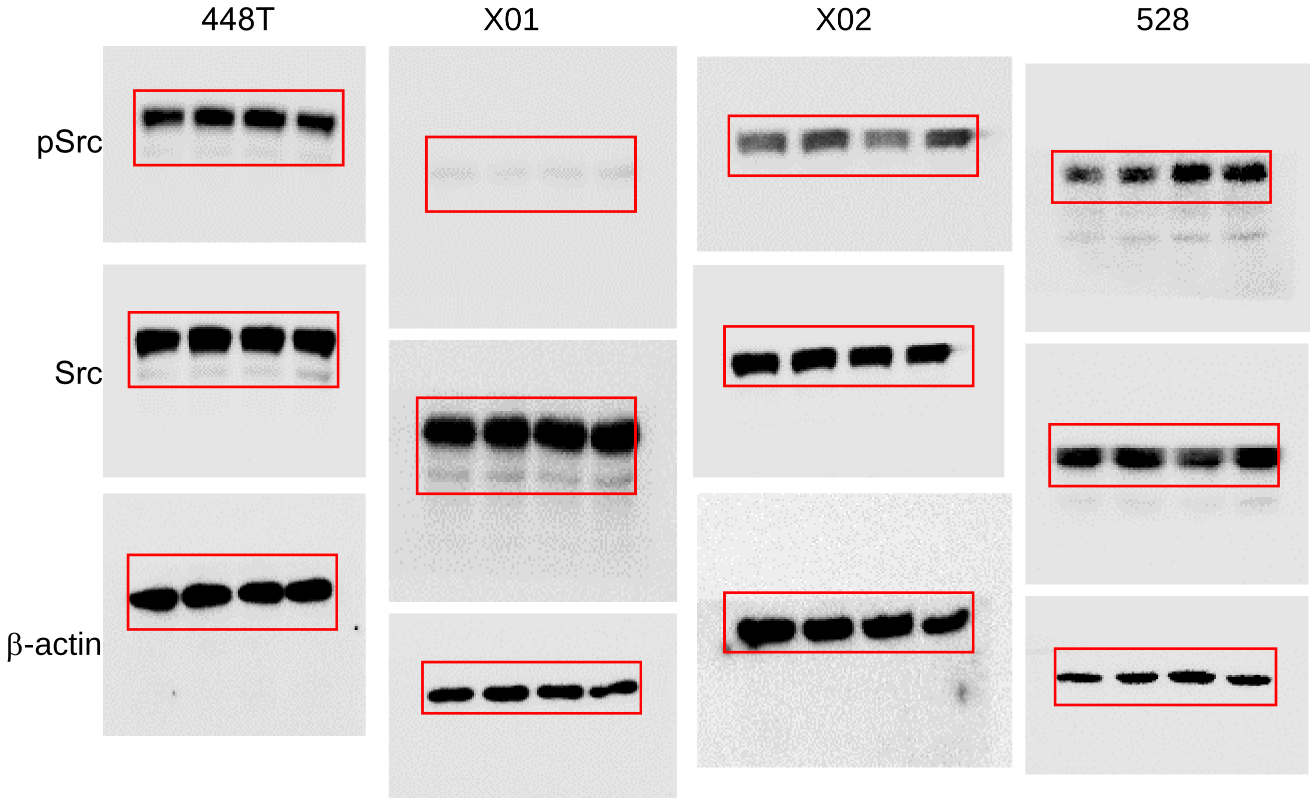

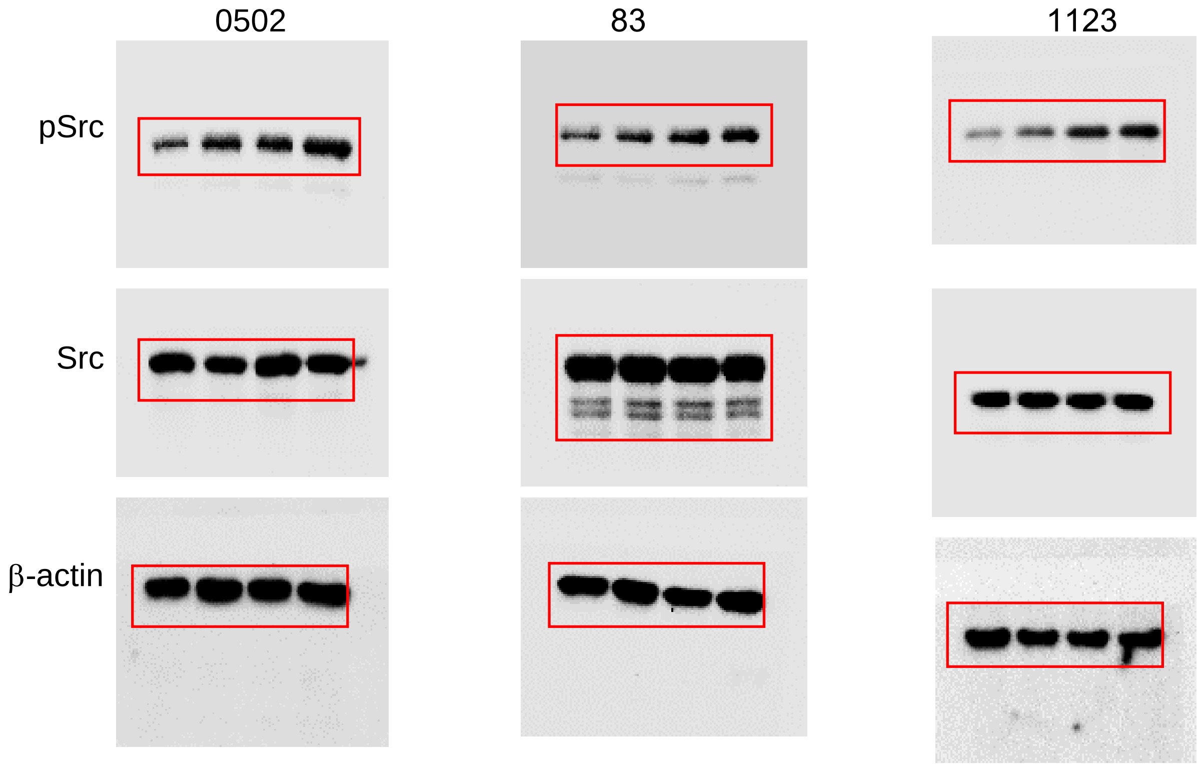

Supplementary Figure 9. Uncropped blots for immunoblot assay

Figure 3d

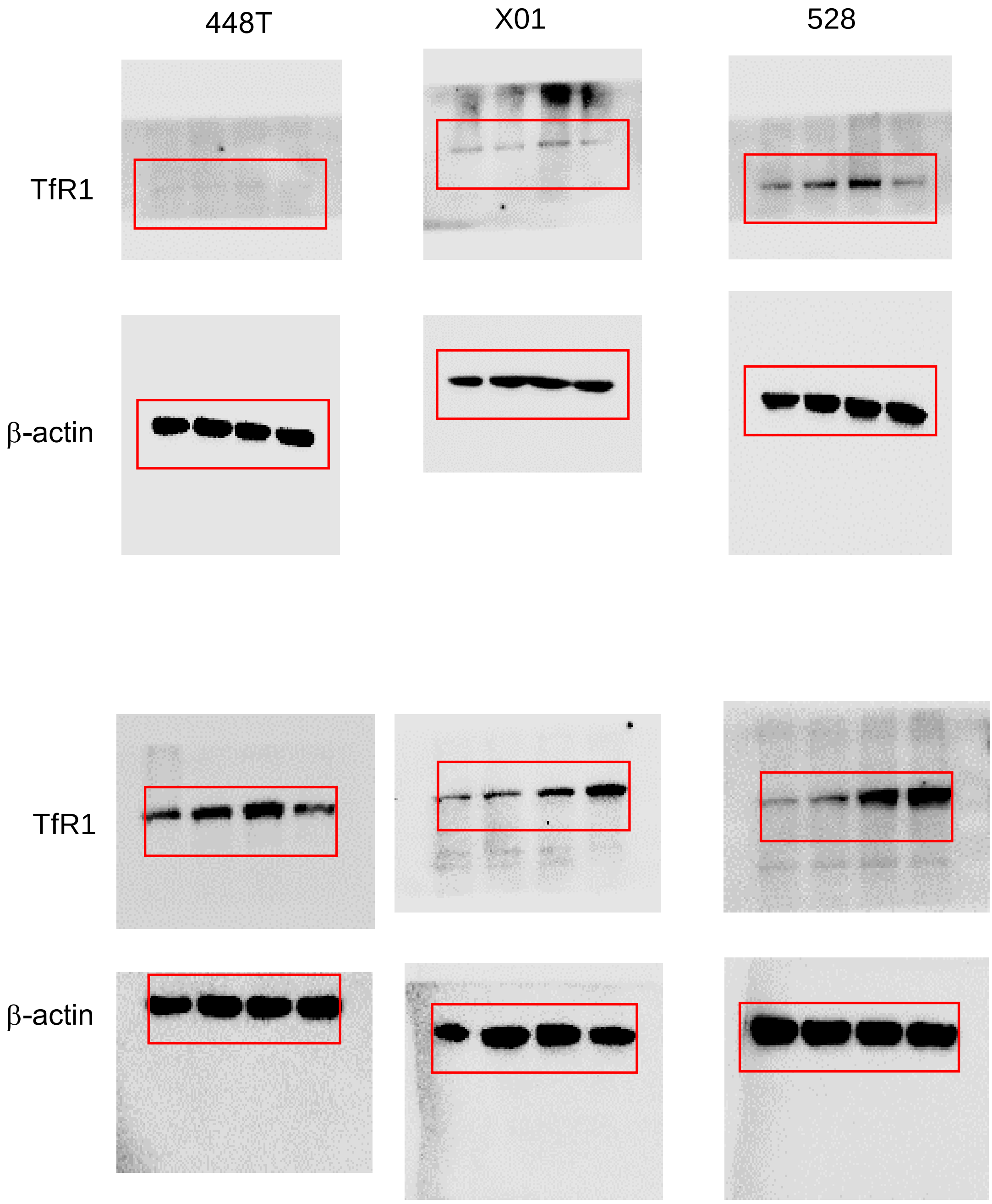

Supplementary Figure 9. Uncropped blots for immunoblot assay

Figure 3f-upper

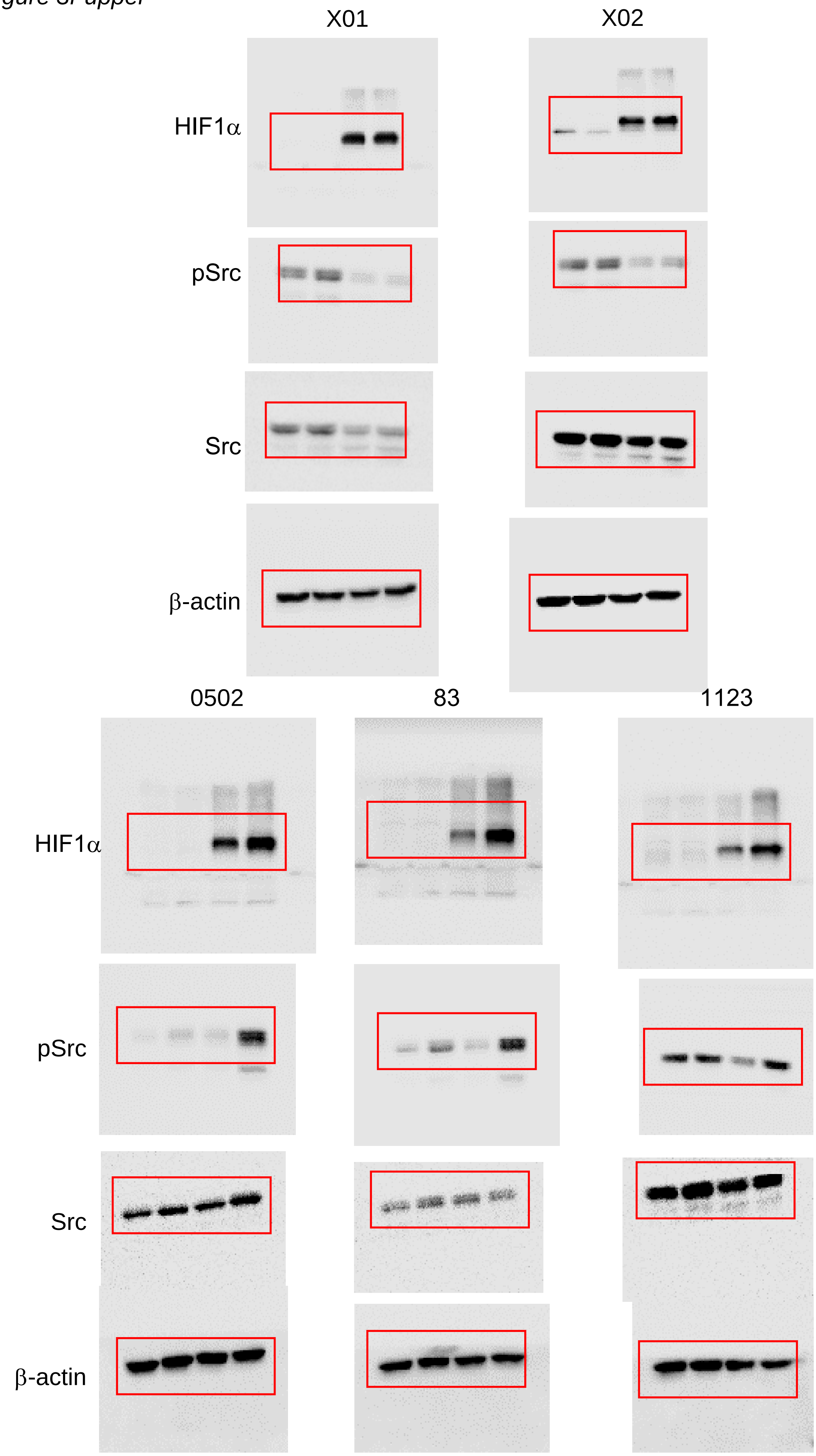

Supplementary Figure 9. Uncropped blots for immunoblot assay

Figure 3f-lower

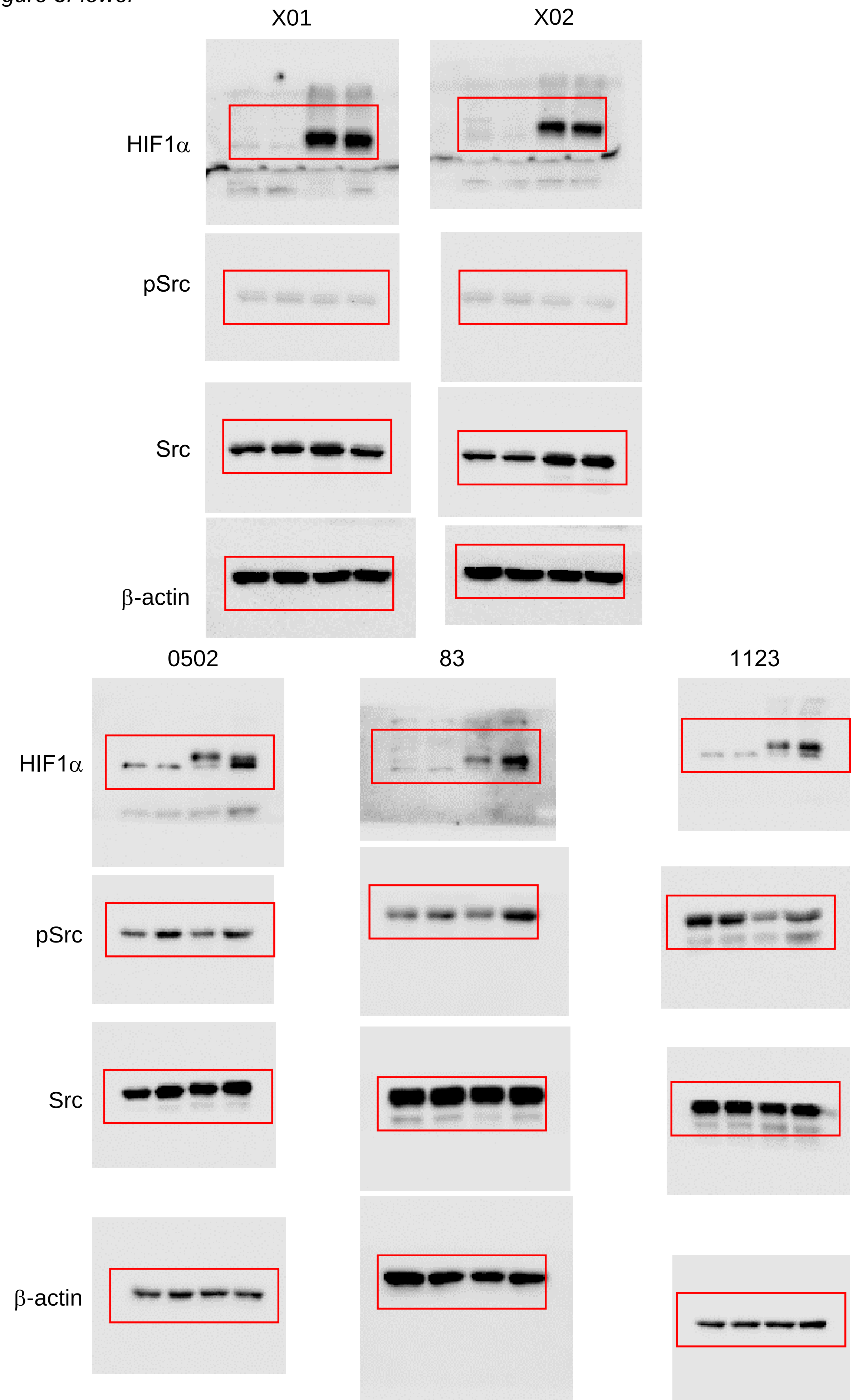

Supplementary Figure 9. Uncropped blots for immunoblot assay

Figure 4a

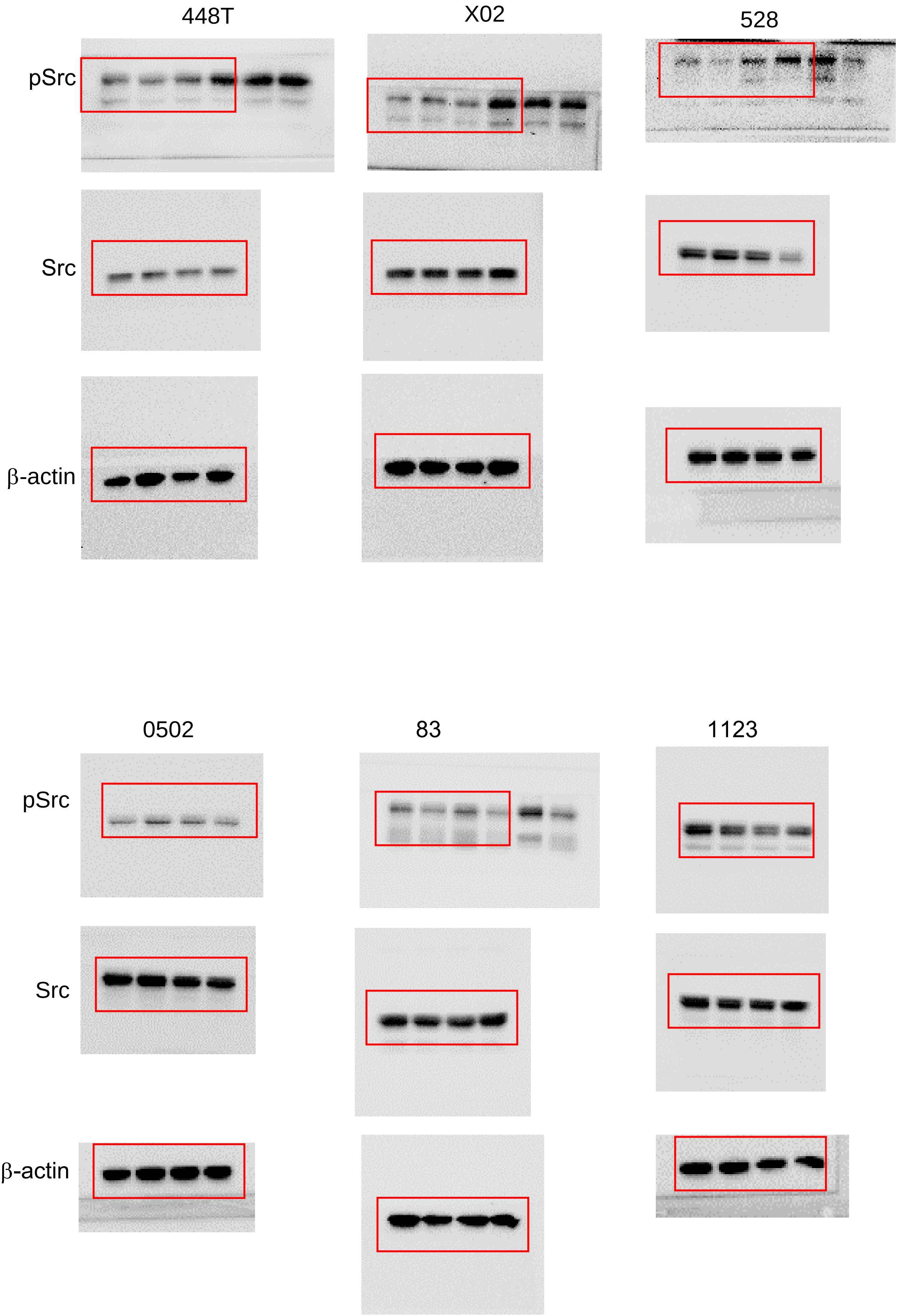

Supplementary Figure 9. Uncropped blots for immunoblot assay

Figure 4d

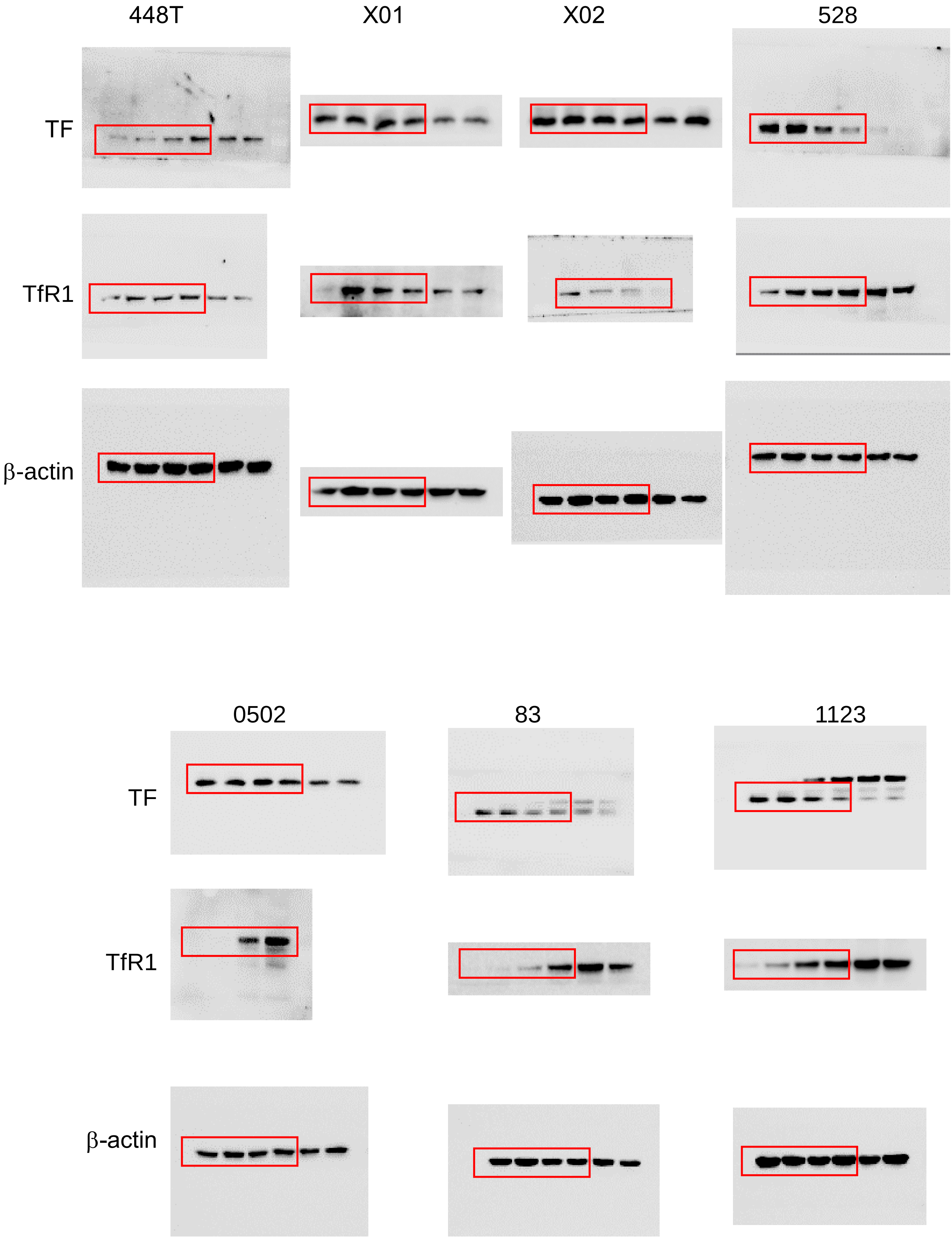

Supplementary Figure 9. Uncropped blots for immunoblot assay

Vo et al.

Supplementary figure 1f

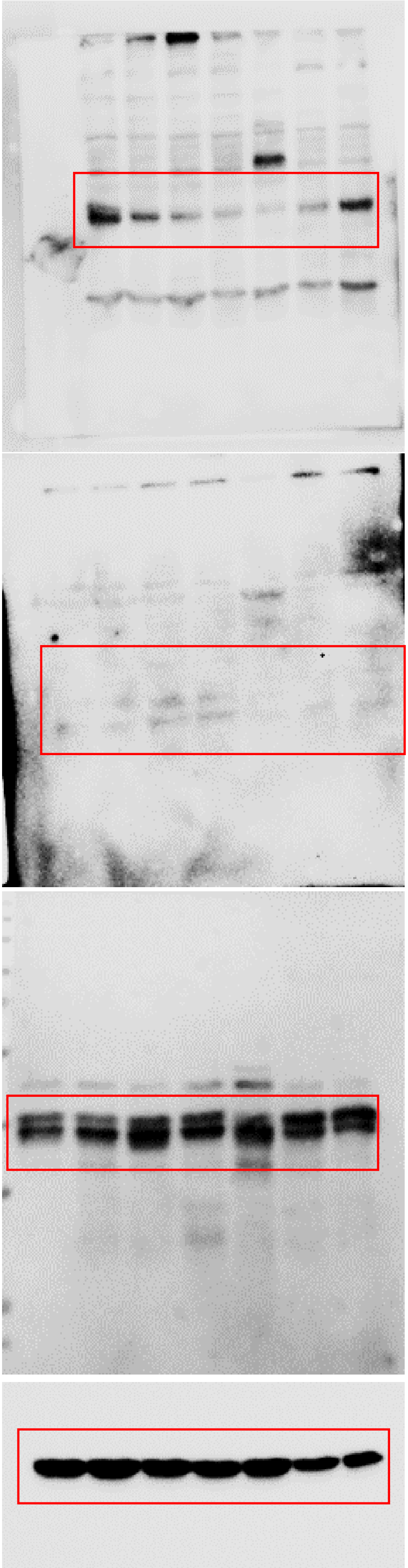

Supplementary figure 1g

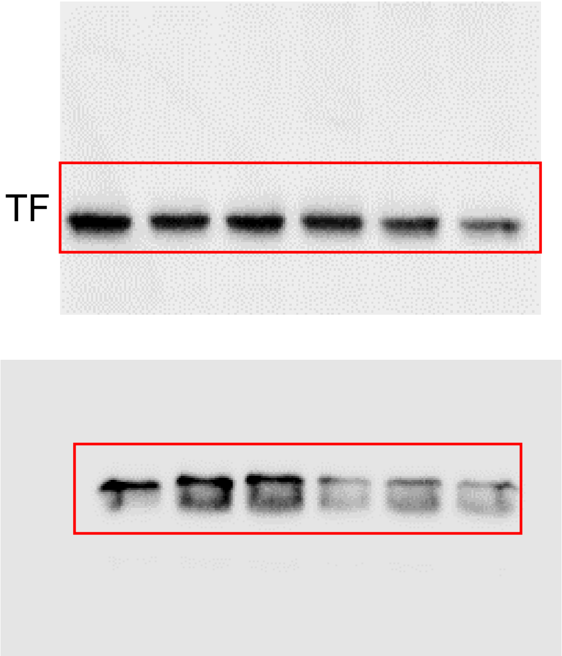

Supplementary Figure 9. Uncropped blots for immunoblot assay

Supplementary figure 3a

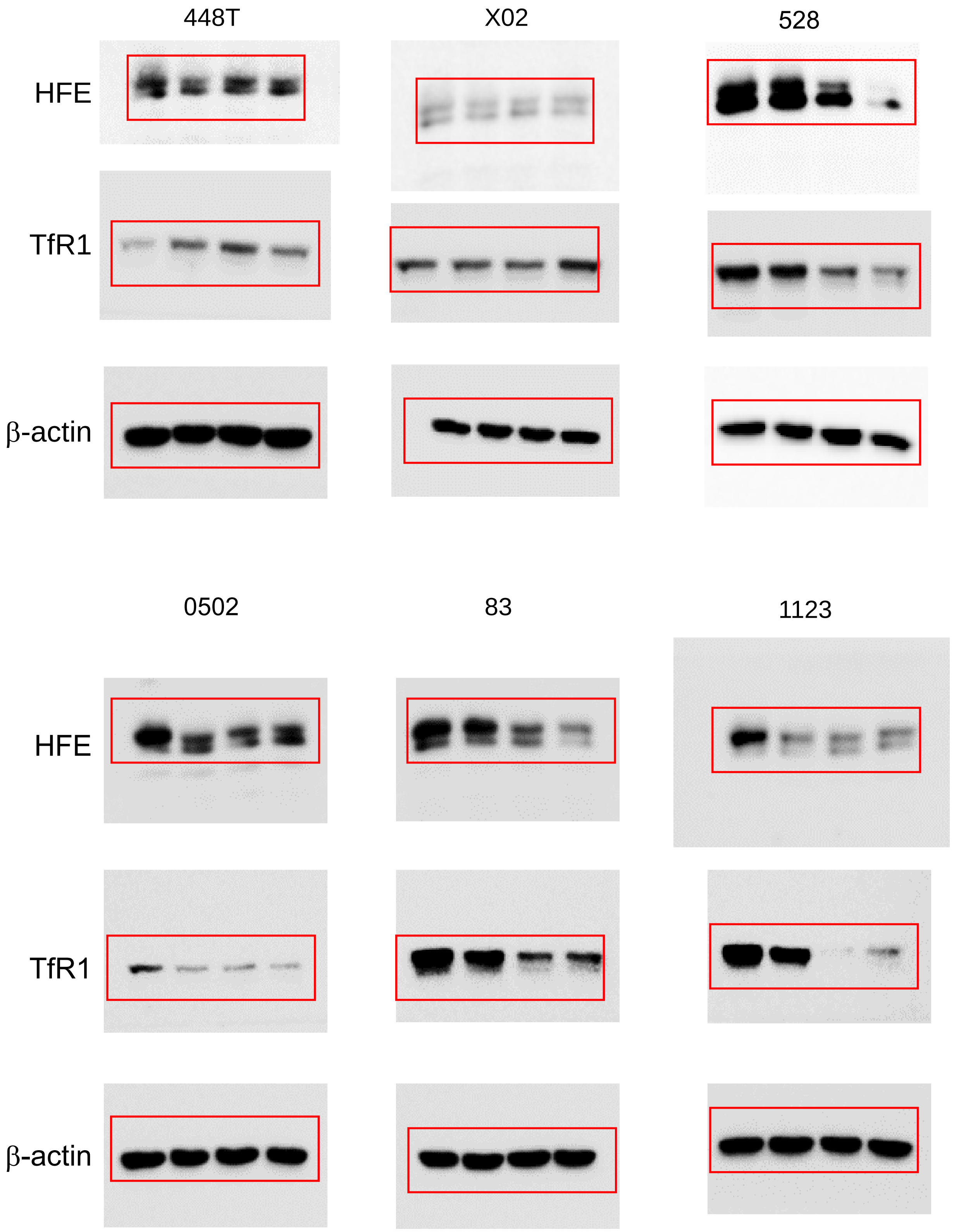

Supplementary Figure 9. Uncropped blots for immunoblot assay

Vo et al.

Supplementary figure 3b

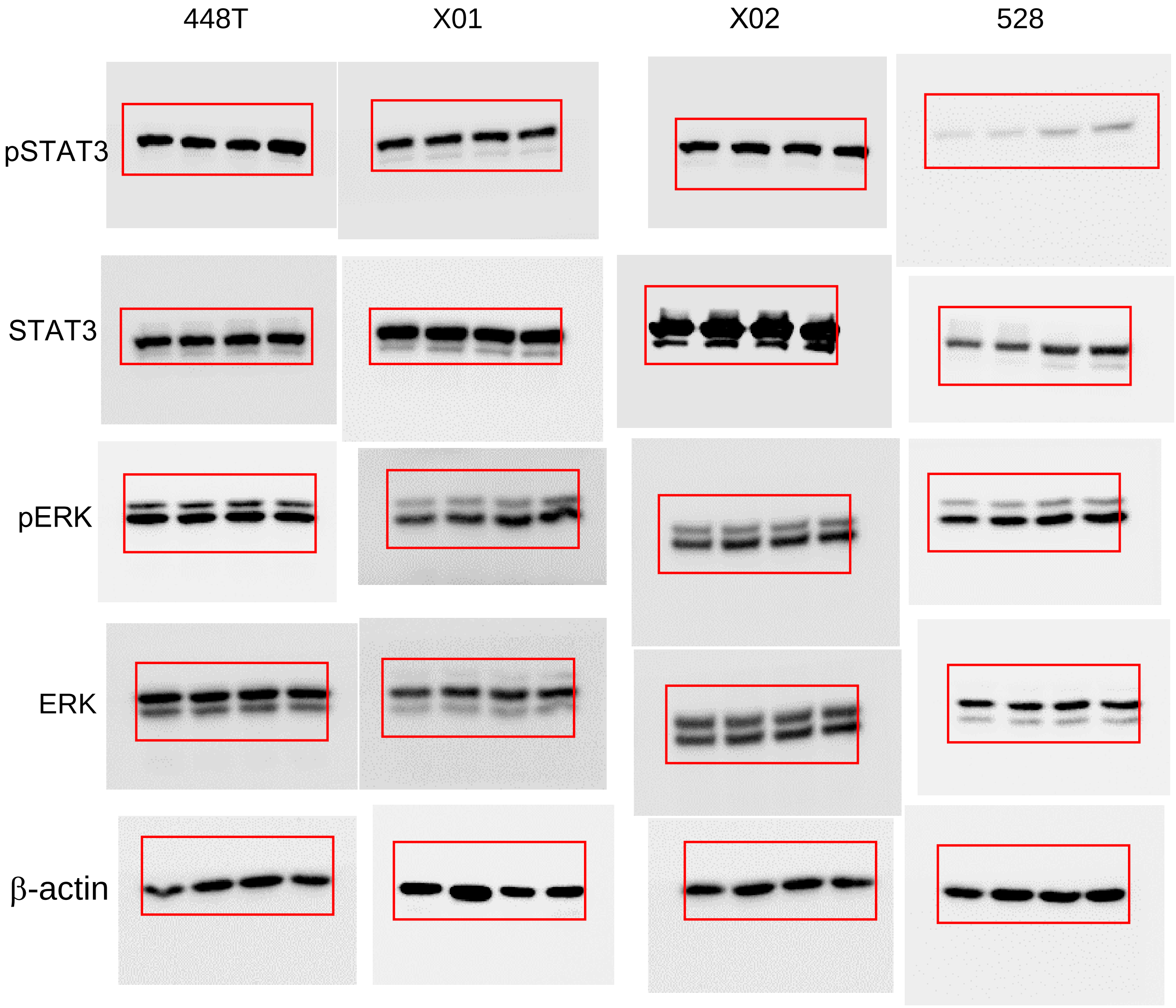

Supplementary Figure 9. Uncropped blots for immunoblot assay

Vo et al.

Supplementary figure 3b

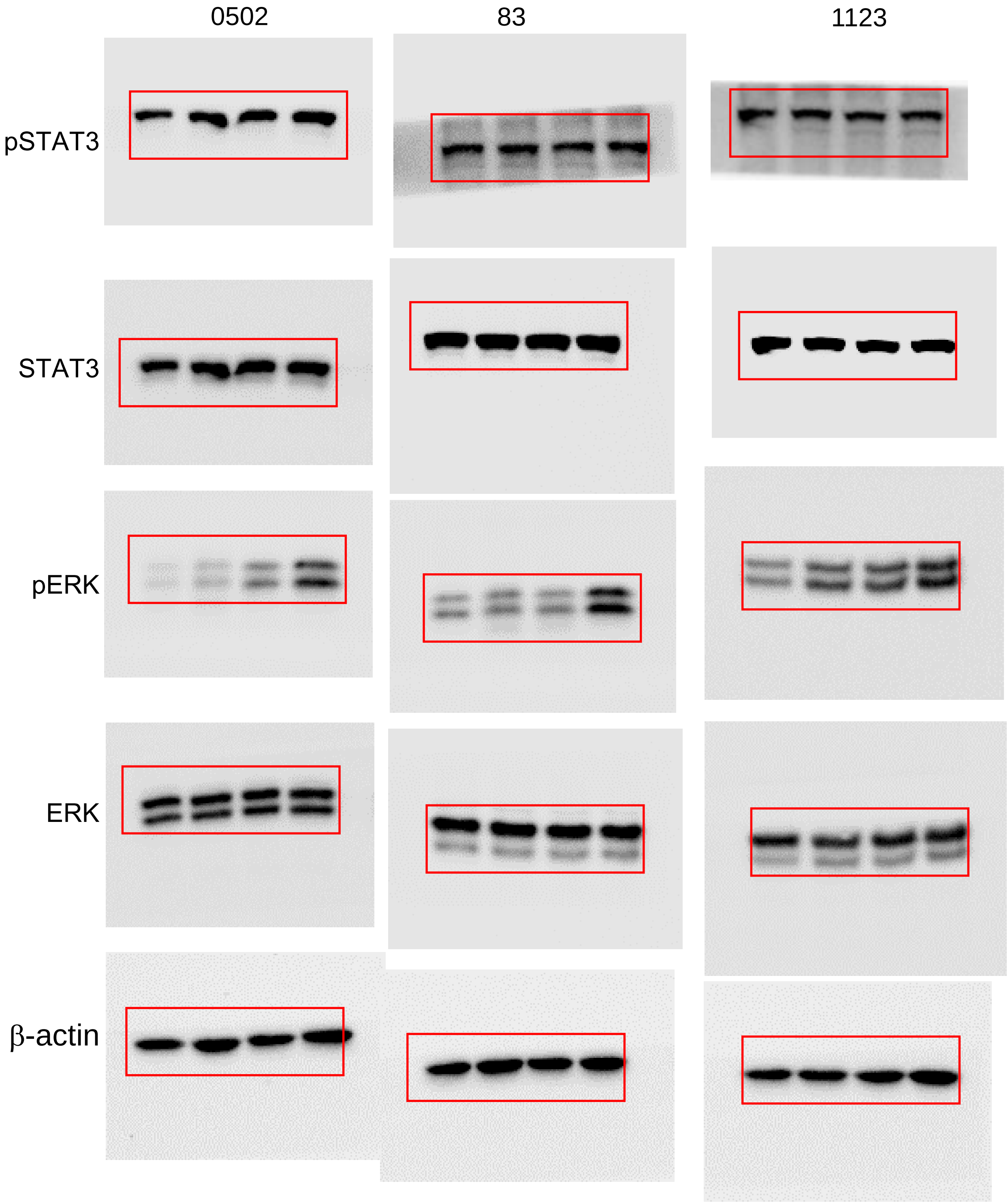

Supplementary Figure 9. Uncropped blots for immunoblot assay

Supplementary figure 3d

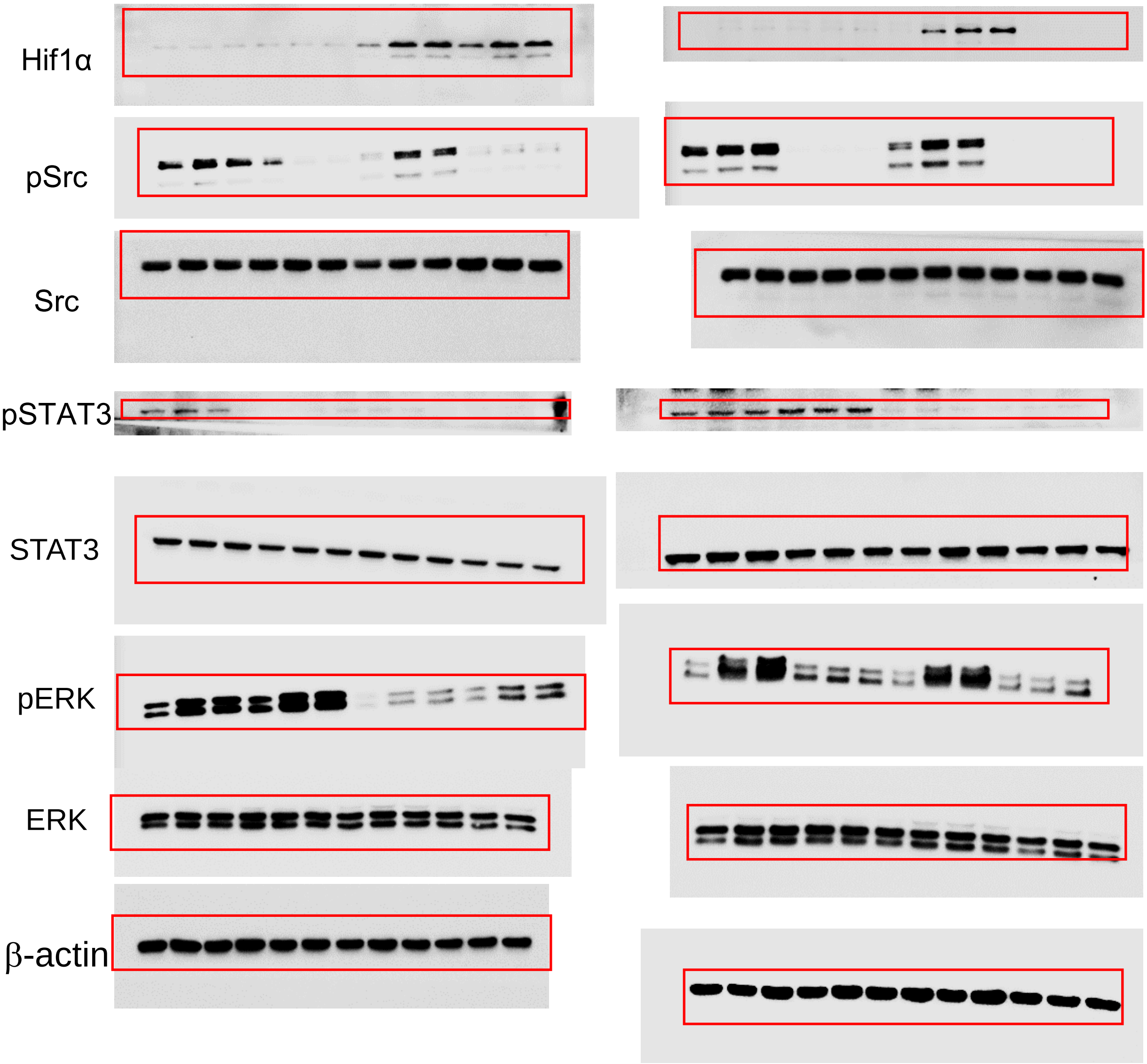

Supplementary Figure 9. Uncropped blots for immunoblot assay

Supplementary figure 4a

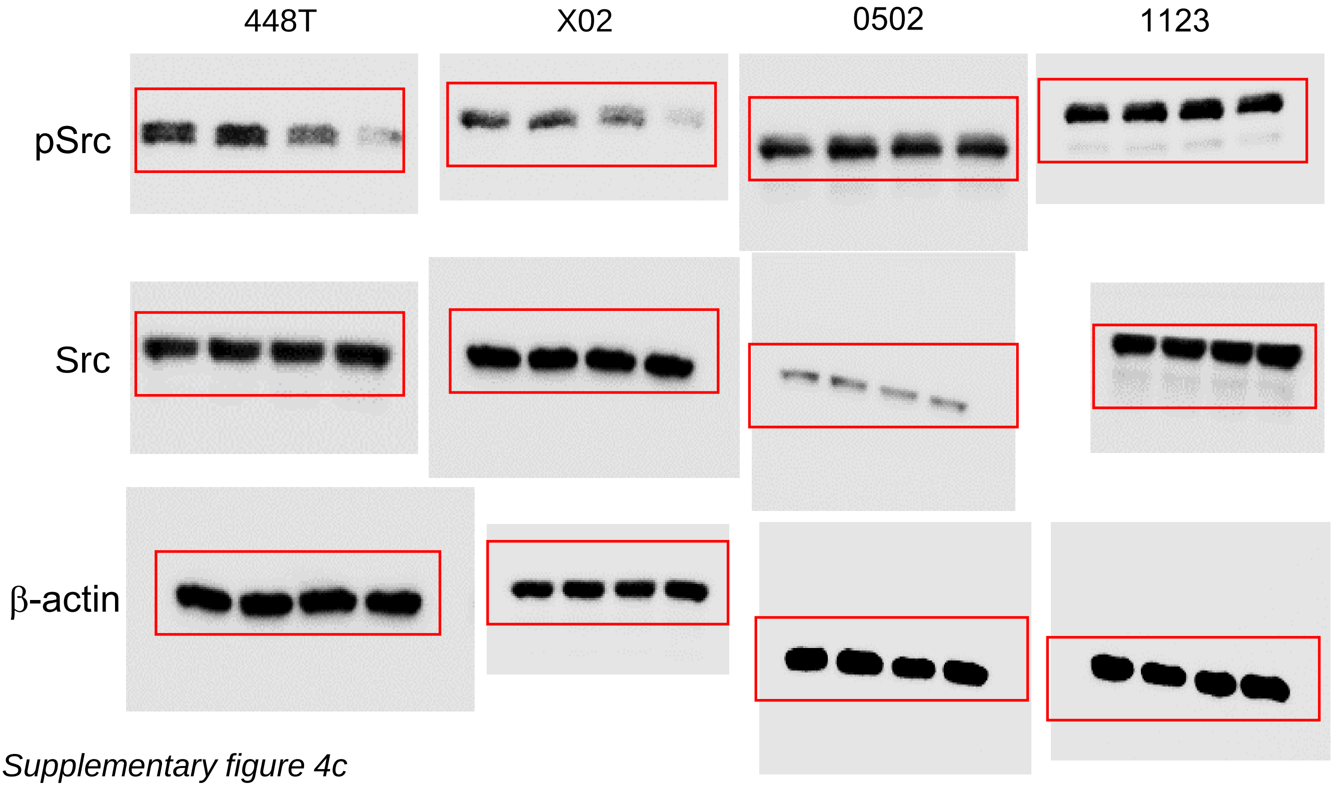

Supplementary figure 4c

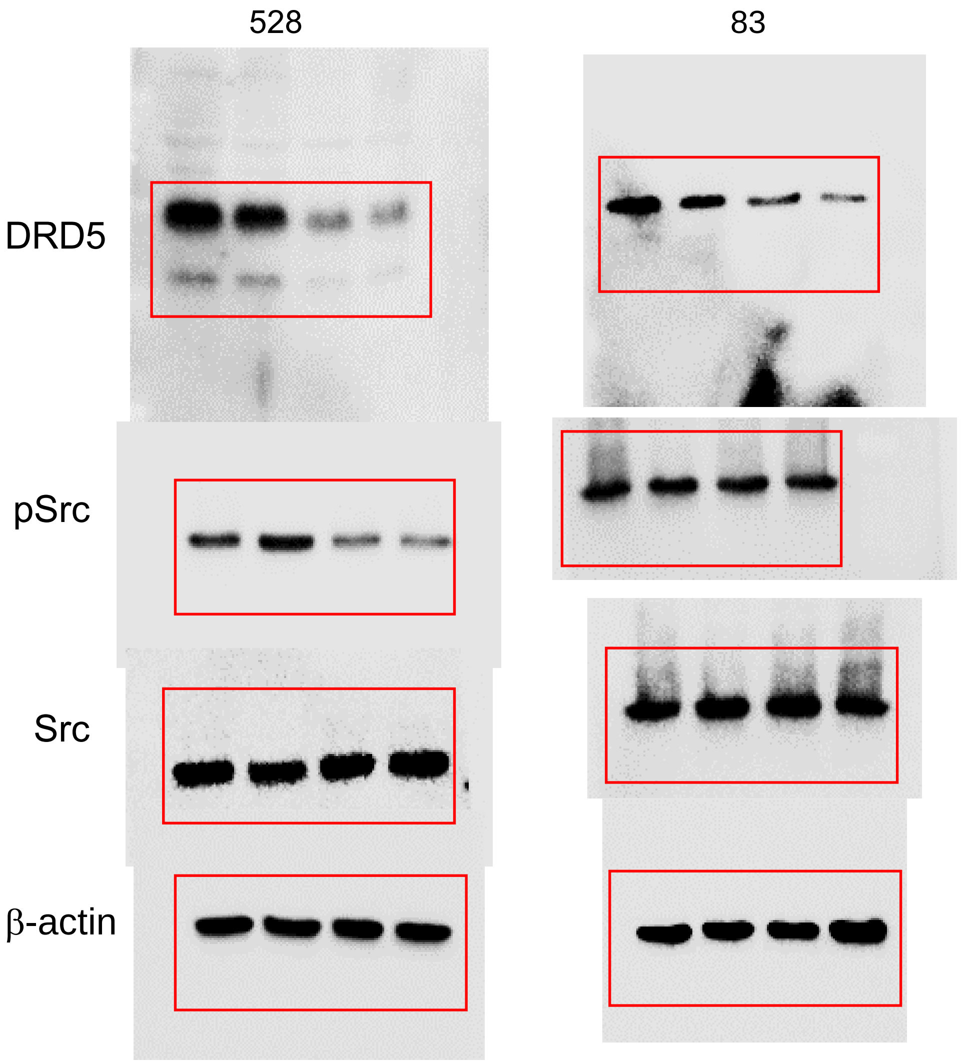

Supplementary Figure 9. Uncropped blots for immunoblot assay

Supplementary figure 5f

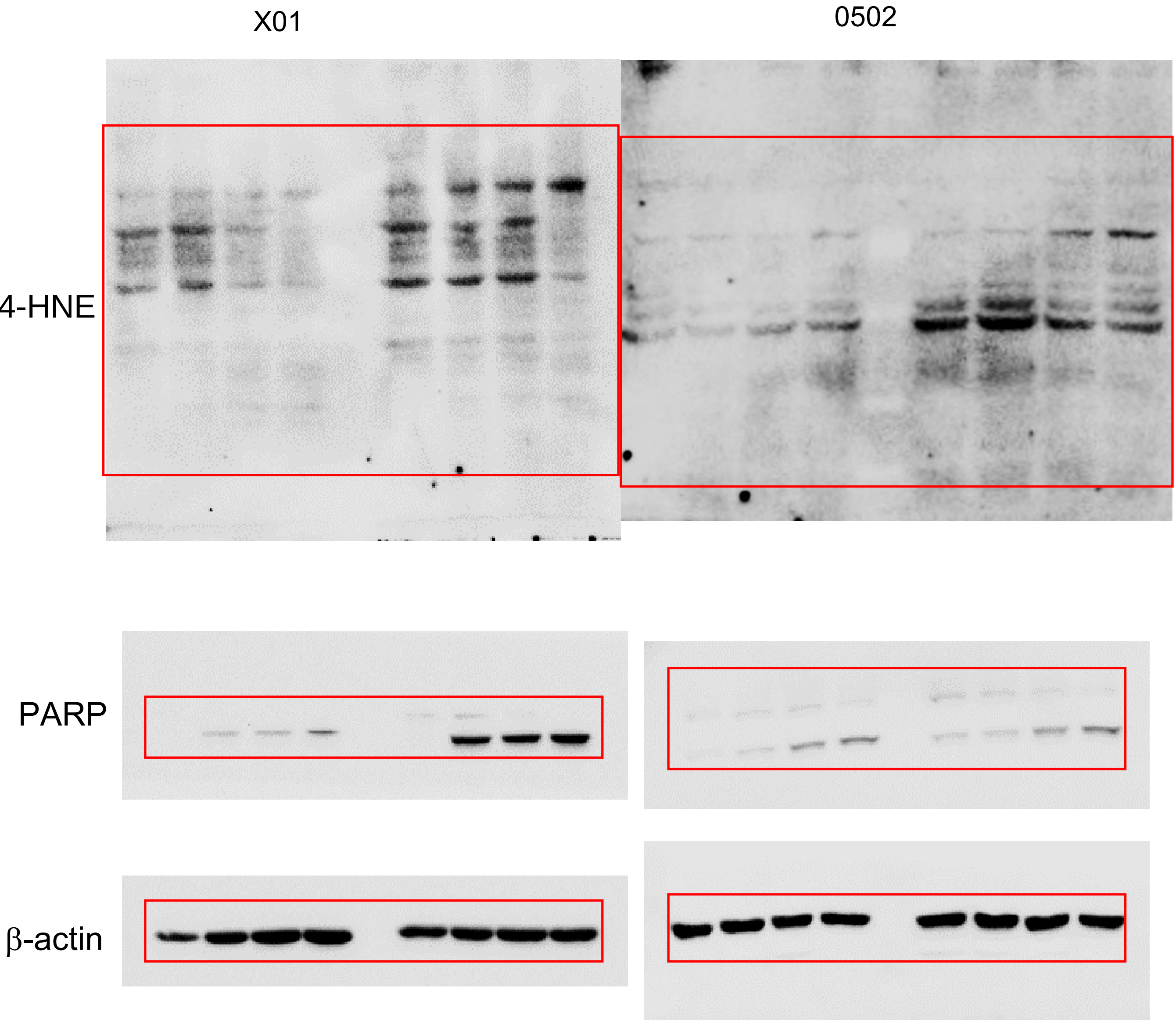

Supplementary figure 7c

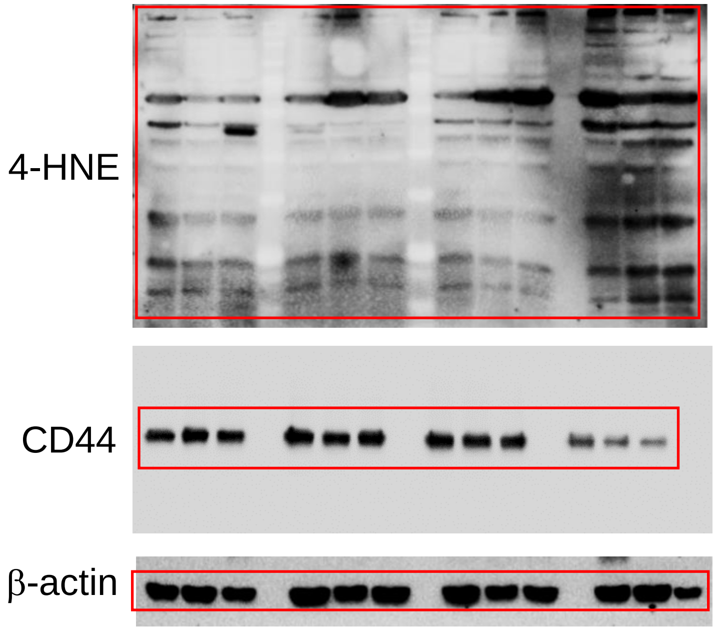

Supplementary Figure 9. Uncropped blots for immunoblot assay

Table S1. List of primer sequences of genes of interest for qPCR

Gene name	Sequence (5' – 3')
18S	Forward: ACCGCAGCTAGGAATAATGGA<br>Reverse: GCCTCAGTTCCGAAAACCA
VEGFA	Forward: CGAGGGCCTGGAGTGTGT<br>Reverse: ATCCGCATAATCTGCATGGT
SLC2A1	Forward: CTGGCATCAACGCTGTCTTC<br>Reverse: GTTGACGATACCGGAGGCAA
DEC1	Forward: CGGAGACCTACCAGGGATGTA<br>Reverse: GGTGCGGCAATTTGTAGGTC
TFRC	Forward: ACCGGCACCATCAAGCT<br>Reverse: TGATCACGCCAGACTTTGC
TF	Forward: TACATAGCGGGCAAGTGTGG<br>Reverse: AGCAAAATACCCTGCCTCTGG
FTL	Forward: TAAAACCCCAGACGCCATGA<br>Reverse: TCCAGGAAGTCACAGAGATGG
FTH1	Forward: GACCCCCATTTGTGTGACTTC<br>Reverse: ATTCCGCCAAGCCAGATTCG
SLC11A2	Forward: GGCTTTGTGTTCTACTTGGGTTG<br>Reverse: GAGATGCTTACCGTATGCCC
SLC40A1	Forward: ACACAAAGATACTGAGCCAAAACC<br>Reverse: TCCATCTCGGAAGGTACGGA
