# Supplementary material for: Iron commensalism of mesenchymal glioblastoma promotes ferroptosis susceptibility upon dopamine treatment
Source: Commun Biol. 2022 Jun 16;5:593. doi: 10.1038/s42003-022-03538-y (PMC9203457; doi:10.1038/s42003-022-03538-y)
Supplement: Supplementary file 5 — Reporting Summary [file 42003_2022_3538_MOESM5_ESM.pdf]

nature portfolio

Corresponding author(s): Yangsik Jeong

Last updated by author(s): Apr 29, 2022



# Reporting Summary

Nature Portfolio wishes to improve the reproducibility of the work that we publish. This form provides structure for consistency and transparency in reporting. For further information on Nature Portfolio policies, see our Editorial Policies and the Editorial Policy Checklist.

## Statistics

For all statistical analyses, confirm that the following items are present in the figure legend, table legend, main text, or Methods section.

| n/a | Confirmed | |
|---|---|---|
| ☒ | ☐ | The exact sample size ($n$) for each experimental group/condition, given as a discrete number and unit of measurement |
| ☒ | ☐ | A statement on whether measurements were taken from distinct samples or whether the same sample was measured repeatedly |
| ☐ | ☒ | The statistical test(s) used AND whether they are one- or two-sided<br>*Only common tests should be described solely by name; describe more complex techniques in the Methods section.* |
| ☒ | ☐ | A description of all covariates tested |
| ☒ | ☐ | A description of any assumptions or corrections, such as tests of normality and adjustment for multiple comparisons |
| ☐ | ☒ | A full description of the statistical parameters including central tendency (e.g. means) or other basic estimates (e.g. regression coefficient) AND variation (e.g. standard deviation) or associated estimates of uncertainty (e.g. confidence intervals) |
| ☐ | ☒ | For null hypothesis testing, the test statistic (e.g. $F$, $t$, $r$) with confidence intervals, effect sizes, degrees of freedom and $P$ value noted<br>*Give P values as exact values whenever suitable.* |
| ☒ | ☐ | For Bayesian analysis, information on the choice of priors and Markov chain Monte Carlo settings |
| ☒ | ☐ | For hierarchical and complex designs, identification of the appropriate level for tests and full reporting of outcomes |
| ☒ | ☐ | Estimates of effect sizes (e.g. Cohen's $d$, Pearson's $r$), indicating how they were calculated |

*Our web collection on statistics for biologists contains articles on many of the points above.*

## Software and code

Policy information about availability of computer code

| | |
|---|---|
| Data collection | No software was used for data collection. |
| Data analysis | GraphPad Prism software version 7.0 (GraphPad Software, La Jolla, CA, USA) and Excel software were used for statistical analysis. |

For manuscripts utilizing custom algorithms or software that are central to the research but not yet described in published literature, software must be made available to editors and reviewers. We strongly encourage code deposition in a community repository (e.g. GitHub). See the Nature Portfolio guidelines for submitting code & software for further information.

## Data

Policy information about availability of data

All manuscripts must include a data availability statement. This statement should provide the following information, where applicable:

- Accession codes, unique identifiers, or web links for publicly available datasets
- A description of any restrictions on data availability
- For clinical datasets or third party data, please ensure that the statement adheres to our policy

The data shown in:
- Figure 1d (upper) was an RNA-seq data previously published by our group (Exp Mol Med. 2020 Apr;52(4):629-642)
- Figure 1d (middle, lower) and supplementary figure 1c, d, e were analyzed from Ivy Glioblastoma Atlas Project (Ivy GAP, http://glioblastoma.alleninstitute.org)
- Figure 5e was The Cancer Genome Atlas (TCGA) glioblastoma data (Nature 2008) obtained from cBioportal for Cancer Genomics (https://www.cbioportal.org/)
- Supplementary figure 2b,c was the web-based gene expression analysis available on http://gliovis.bioinfo.cnio.es/
- Supplementary figure 8 was the web-based survival analysis using data from GSE30472, GSE42669, GSE4412, GSE7696, Chinese Glioma Genome Atlas (CCGA), and TCGA (Nature 2008)

# Field-specific reporting

Please select the one below that is the best fit for your research. If you are not sure, read the appropriate sections before making your selection.

☒ Life sciences ☐ Behavioural & social sciences ☐ Ecological, evolutionary & environmental sciences

For a reference copy of the document with all sections, see nature.com/documents/nr-reporting-summary-flat.pdf

# Life sciences study design

All studies must disclose on these points even when the disclosure is negative.

| | |
|---|---|
| Sample size | The sample size for individual experiments was decided based on the aspects of cost, time, and convenience. The strategy was to: 1. meet the requirement for statistical analysis power, 2. reduce unnecessary and wasting usage of samples. In the experiments using human tissues, the number of samples was also dependent on accessibility. |
| Data exclusions | No data was excluded from analysis. |
| Replication | Different experiment designs and protocols were used to verify the replication of the data. |
| Randomization | The human glioblastoma tissues from two different anatomical regions were not picked up randomly but rather on purpose of choosing center and margin region of the tumor. |
| Blinding | The human glioblastoma tissues from two different anatomical regions were recognized by investigators because they were decided prior to the experiments and visually different in shape. |

# Reporting for specific materials, systems and methods

We require information from authors about some types of materials, experimental systems and methods used in many studies. Here, indicate whether each material, system or method listed is relevant to your study. If you are not sure if a list item applies to your research, read the appropriate section before selecting a response.

## Materials & experimental systems

| n/a | Involved in the study | |
|---|---|---|
| ☐ | ☒ | Antibodies |
| ☐ | ☒ | Eukaryotic cell lines |
| ☒ | ☐ | Palaeontology and archaeology |
| ☐ | ☒ | Animals and other organisms |
| ☐ | ☒ | Human research participants |
| ☒ | ☐ | Clinical data |
| ☒ | ☐ | Dual use research of concern |

## Methods

| n/a | Involved in the study | |
|---|---|---|
| ☒ | ☐ | ChIP-seq |
| ☒ | ☐ | Flow cytometry |
| ☒ | ☐ | MRI-based neuroimaging |

## Antibodies

| | |
|---|---|
| Antibodies used | Transferrin (Santa Cruz, sc-374441), TfR1 (Santa Cruz, sc-32272), DRD2 (Santa Cruz, sc-5303), DRD3 (Santa Cruz, sc-136170), DRD4 (Santa Cruz, sc-136169), DRD5 (Santa Cruz, sc-376088), TH (Santa Cruz, sc-25269), PAH (Santa Cruz, sc-271258), and DDC (Santa Cruz, sc-293287), SOX2 (Cell Signaling Technology, #3728), CD44 (Cell Signaling Technology, #3570), pSrc (Cell Signaling Technology, #6943), Src (Cell Signaling Technology, #2108), pSTAT3 (Cell Signaling Technology, #9131), STAT3 (Cell Signaling Technology, #9139), pERK (Cell Signaling Technology, #9101S), ERK (Cell Signaling Technology, #9102S), PARP (Cell Signaling Technology, #9542S), HIF1α (Novus Biologicals, NB100-449), DRD1 (Novus Biologicals, NBP2-66807); 4-HNE (Abcam, ab46545), β-actin (Abcam, ab6276), and HFE (Abcam , ab133369), HRP conjugated anti-mouse IgG (Abcam,ab6728), and anti-rabbit IgG (Innovative Research, G-21234). |
| Validation | The antibodies were validated based on manufacturers' datasheet. Some of the antibodies have been widely used in our lab for years. |

## Eukaryotic cell lines

Policy information about cell lines

| | |
|---|---|
| Cell line source(s) | The glioblastoma cell lines 448T, X01, X02, 528, 83, and 1123 were kindly provided by Jong Bae Park (National Cancer Center, South Korea), and cell line 0502 was kindly provided by Myung-Jin Park (KIRAM, South Korea), as previously described by our lab (Exp Mol Med. 2020 Apr;52(4):629-642). For revision experiments, we have used U937 and THP-1 macrophage cells provided by Mee-Yon Cho and Kyu-sang Park (Yonsei University Wonju College of Medicine, South Korea). |

| | |
|---|---|
| Authentication | None of the cell lines used was authenticated. |
| Mycoplasma contamination | The cell lines were not tested for mycoplasma contamination. |
| Commonly misidentified lines (See ICLAC register) | N/A |

## Animals and other organisms

Policy information about studies involving animals; ARRIVE guidelines recommended for reporting animal research

| | |
|---|---|
| Laboratory animals | 5-weeks-old female Balb/c nude mice |
| Wild animals | This study did not use any wild animals. |
| Field-collected samples | This study did not use any field-collected samples. |
| Ethics oversight | Animal experiments were performed under the approval of Institutional Animal Care and Use Committee (IACUC) of Yonsei University Wonju College of Medicine (Approval number: YWC-170907-3). |

Note that full information on the approval of the study protocol must also be provided in the manuscript.

## Human research participants

Policy information about studies involving human research participants

| | |
|---|---|
| Population characteristics | We have used human tissue from glioblastoma patients obtained from Yonsei University Wonju Severance Christian Hospital. |
| Recruitment | Wonju Severance Christian Hospital has recruited the glioblastoma patients. |
| Ethics oversight | The study was approved by the Committee of Institutional Review Board of Yonsei University Wonju Severance Christian Hospital (Approval number: CR320372). |

Note that full information on the approval of the study protocol must also be provided in the manuscript.
